# Association between women’s household decision-making autonomy and health insurance enrollment in sub-saharan Africa

**DOI:** 10.1186/s12889-023-15434-z

**Published:** 2023-03-30

**Authors:** Betregiorgis Zegeye, Dina Idriss-Wheeler, Bright Opoku Ahinkorah, Edward Kwabena Ameyaw, Abdul-Aziz Seidu, Nicholas Kofi Adjei, Sanni Yaya

**Affiliations:** 1 HaSET Maternal and Child Health Research Program, Shewarobit Field Office, Shewarobit, Ethiopia; 2 Interdisciplinary School of Health Sciences, University of Ottawa, Ottawa, Canada; 3 School of Public Health, Faculty of Health, University of Technology Sydney, Ultimo, Australia; 4 Lingnan University Graduate School, Tuen Mun, Hong Kong; 5 Centre for Gender and Advocacy, Takoradi Technical University, P.O.Box 256, Takoradi, Ghana; 6 College of Public Health, Medical and Veterinary Sciences, James Cook University, QLD4811 Townsville, Queensland Australia; 7 Department of Public Health, Policy and Systems, University of Liverpool, Liverpool, UK; 8 School of International Development and Global Studies, University of Ottawa, 120 University Private, K1N 6N5 Ottawa, ON Canada; 9 The George Institute for Global Health, Imperial College London, London, UK

**Keywords:** Women’s decision-making autonomy, Health insurance, Universal health coverage, DHS, Sub-saharan Africa, Global health

## Abstract

**Background:**

Out of pocket payment for healthcare remains a barrier to accessing health care services in sub-Saharan Africa (SSA). Women’s decision-making autonomy may be a strategy for healthcare access and utilization in the region. There is a dearth of evidence on the link between women’s decision-making autonomy and health insurance enrollment. We, therefore, investigated the association between married women’s household decision making autonomy and health insurance enrollment in SSA.

**Methods:**

Demographic and Health Survey data of 29 countries in SSA conducted between 2010 and 2020 were analyzed. Both bivariate and multilevel logistic regression analyses were carried out to investigate the relationship between women’s household decision-making autonomy and health insurance enrollment among married women. The results were presented as an adjusted odds ratio (AOR) and the 95% confidence interval (CI).

**Results:**

The overall coverage of health insurance among married women was 21.3% (95% CI; 19.9-22.7%), with the highest and lowest coverage in Ghana (66.7%) and Burkina Faso (0.5%), respectively. The odds of health insurance enrollment was higher among women who had household decision-making autonomy (AOR = 1.33, 95% CI; 1.03–1.72) compared to women who had no household decision-making autonomy. Other covariates such as women’s age, women’s educational level, husband’s educational level, wealth status, employment status, media exposure, and community socioeconomic status were found to be significantly associated with health insurance enrollment among married women.

**Conclusion:**

Health insurance coverage is commonly low among married women in SSA. Women’s household decision-making autonomy was found to be significantly associated with health insurance enrollment. Health-related policies to improve health insurance coverage should emphasize socioeconomic empowerment of married women in SSA.

## Background

The 2030 Sustainable Development Goals (SDGs) emphasize that every individual must have access to quality health services without financial hardship [1]. Since the adoption of the SDGs, there has been a renewed interest in low-and middle-income countries (LMICs) to increase efforts towards universal health coverage to ensure equal access to quality health care [2]. More specifically, SDG Goal 3.8 is dedicated to protecting people from the financial risks of catastrophic health care expenditures by minimizing individuals’ out of pocket healthcare-related costs; this has the broader goal of improving population health and promoting socioeconomic well-being and national development [2–4]. Estimates by the World Health Organization and the World Bank, show that in 2010, 179 million people worldwide (2.6% of the population) suffered catastrophic healthcare payments greater than 25% of total income or consumption [5]. These estimates revealed that the African region had the fastest increase in catastrophic payments [5].

In LMICs, more than 150 million people endure out of pocket costs related to health-related diseases. Furthermore, over two-thirds suffer from chronic poverty and related problems [6, 7]. While health expenditure per capita in sub-Saharan Africa (SSA) has increased by an annualized 3.2% over the last two decades (1995–2014) [8], approximately 36% of healthcare spending in the region continues to be made through direct out of pocket payments, compared to only 22% for the rest of the world [9]. Moreover, existing evidence indicates that catastrophic health expenditures within SSA vary widely - ranging from 1% in Botswana to 25% in Nigeria [10, 11]. Unfortunately, out of pocket financing for healthcare is the main approach to payment for healthcare in SSA, leading to low utilization of and access to healthcare services [5, 12–14].

There are inequitable impacts of out of pocket payments on vulnerable sub-groups in LMICs which deters both seeking of and access to healthcare services, and leads to unmet health care needs and inequities. This is especially true for married women, whose autonomy to seek or access health services, make decisions about their own healthcare or how to spend household or personal income, is complicated by patriarchal gender and cultural norms [15–17]. Challenges of traditional male authority and control in a marriage lead to tension, conflict, and higher likelihood of domestic violence [18, 19]. Subsequently, the ability to seek or utilize health care facilities is linked to women’s household decision-making autonomy [20]. A recent study in Nigeria by Ifelunini et al. suggested that autonomy in household decisions (i.e., deciding on women’s income and household healthcare services expenditures) increased the likelihood for uptake of maternal healthcare services [21]. Alternatively, when the husband/partner makes the sole or joint decision, the demand is reduced. One such way is having health insurance. Ensuring that women have access to health insurance is one effective way to mitigate the power imbalances that exist due to patriarchal gender and cultural norms.

Another key dimension in seeking and accessing health care is women’s ability to access their own finances, such as employment, income, and health insurance. When investigating issues faced by women in SSA when accessing healthcare, it was found that getting money needed for treatment (50.1%) emerged as the predominant barrier [15]. Indeed, studies have shown that women with access to finances were able to make decisions about seeking health services without consulting others, such as their husband or family members [20, 22].

Since the 2030 Agenda for Sustainable Development, many LMICs have strategically attempted to implement health insurance schemes to provide universal access to health services without undue financial hardships as they work towards universal health coverage [23]. Health Insurance schemes are platforms created and operated by organizations or companies that can be community-based, government-only, or private-for-profit; they offer a shared risk to cover the cost of healthcare services [2]. Ultimately, the purpose of health insurance is to reduce the financial burden of paying out of pocket for health care by pooling resources and sharing the risk of unexpected health events [24–26]. Risk-sharing mechanisms are particularly important in SSA, where most countries devote insufficient resources to healthcare and associated commodities, including medications, consequently resulting in out-of-pocket payments [24–26]. A study by Ataguba and McIntyre demonstrated the regressive impact of out of pocket payments, noting that using progressive health financing mechanisms (i.e., direct taxes and private health insurance medical schemes) allows the poorest quintiles to pay less as a proportion of their ability to pay [27]. Consequently, groups who face healthcare seeking inequities – particularly women - have a higher likelihood of seeking or accessing healthcare with medical insurance schemes. In Nigeria, Ugbor et al. found that women who participated in community health insurance schemes improved health seeking behaviour by 17% compared to women who did not participate [28], demonstrating the progressive impact of having health-insurance.

Given the importance of women’s decision-making autonomy [20] as well as the relevance of health insurance enrollment in accessing or seeking health care in SSA [29], it is important to consider how the two intersect. Several studies have examined health insurance coverage in SSA, investigating inequality in health insurance coverage in addition to the prevalence and factors associated with coverage in urban regions. Some of these studies have focused on health seeking behaviours of reproductive aged women across a number of SSA countries [3, 28, 30–32]. Other literature has also examined women’s decision-making power in the household and its positive influence on the use of health services, particularly in SSA [33, 34]. However, no study to date has assessed the relationship between women’s household decision-making autonomy and health insurance enrollment in SSA. In this study, we investigated determinants, both at the individual/household and community levels, of married women’s health insurance enrollment, with a focus on household decision making autonomy in SSA. Findings from this work will help understand the key determinants of health insurance enrollment, particularly the role of women’s decision-making autonomy, to inform strategies to encourage their participation in health insurance schemes. Additionally, this work focuses on married women who tend to face higher health inequities in seeking or accessing health services. We aim to provide evidence to inform creation of national policies that promote gender equity and women’s empowerment for voiceless and disadvantaged groups, such as women facing domestic violence [20].

## Methods

### Data source

We pooled data from the Demographic and Health Surveys (DHSs) of 29 countries in SSA, conducted between 2010 and 2020. Studied countries were selected based on the availability of the outcome variable and key explanatory variables. The surveys were nationally representative and include data on a wide range of public health related issues including women’s autonomy and health insurance coverage [35]. Details of the sampling procedure and data collection methods are outlined elsewhere [36]. Studied countries were selected based on the availability of the outcome variable (health insurance enrollment), key explanatory variable (women’s household decision-making autonomy) and covariates in their datasets. A total of 226,734 married women in the reproductive age group were included in the analysis. The DHS datasets are available in the public domain and can be accessed at http://dhsprogram.com/data/available-datasets.cfm. Table 1 provides detailed information about selected countries, year of survey, and samples.

**Table 1:** Survey year, included countries and their respective sampled population

Country	Year of survey	Sampled population	
		Weighted number (n)	Weighted %
1. Angola	2015/16	8033	3.5
2. Benin	2017/18	11,170	4.9
3. Burkina Faso	2010	13,392	5.9
4. Burundi	2016/17	9,559	4.2
5. Cameroon	2018/19	7,463	3.3
6. Chad	2014/15	4,607	2.0
7. Comoros	2012	3,291	1.5
8. Congo	2011/12	6,750	3.0
9. Congo DR	2013/14	12,409	5.5
10. Cote d’Ivoire	2011/12	6,411	2.8
11. Ethiopia	2016	9,824	4.3
12. Gabon	2012	4,749	2.1
13. Gambia	2019/20	6,873	3.0
14. Ghana	2014	5,452	2.4
15. Guinea	2018	7,812	3.4
16. Kenya	2014	8,992	4.0
17. Lesotho	2014	3,609	1.6
18. Liberia	2019/20	5,875	2.6
19. Malawi	2015/16	15,952	7.0
20. Mali	2018	8,332	3.7
21. Namibia	2013	3,330	1.5
22. Niger	2012	9,509	4.2
23. Senegal	2010/11	10,804	4.8
24. Sierra Leone	2019	9,837	4.3
25. South Africa	2016	1,359	0.6
26. Togo	2013	6,353	2.8
27. Uganda	2016	11,377	5.0
28. Zambia	2018	7,597	3.4
29. Zimbabwe	2015	6,013	2.7
**Total**		**226,734**	**100.00**

### Study variables

#### Outcome variable

The outcome variable of interest was health insurance status, which included both public and private insurance of participants at the time of interview. If they were covered by either public or private, they were coded “yes” (insured), and individuals not insured were coded “no” [37, 38].

#### Explanatory variable

The explanatory variable was women’s household decision-making autonomy. In the DHS, married women were asked three decision-making questions: who decides about (i) “own (respondent’s) health?” (ii) “large household purchases?” and (iii) “family or relatives’ visits?” These variables were used to create the outcome measure of women’s decision-making autonomy. The variables were coded as binary. Married women who made decisions either alone or together with their husbands on all three aforementioned decision-making parameters were considered as empowered and coded as “1”, whereas married women who did not make decision either alone or together with husband on all three decision-making parameters or made decision on one or two decision-making parameters were considered as not empowered and coded “0”, as conceptualized by some previous studies [39, 40]).

#### Covariates

Based on previous studies [3, 10, 14, 24, 30–32], we identified potential individual/household and community level variables as covariates. The individual level variables were women’s age in years (15-19, 20-24, 25-29, 30-34, 35-39, 40-44, 45-49), women’s educational level (no formal education, primary school, secondary school, higher), husband’s educational level (no formal education, primary school, secondary school, higher) and wealth status (poorest, poorer, middle, richer, richest), currently employed (yes, no). The rest were media exposure (no, yes), sex of household head (male, female), parity (less than 5, 5 and above), media exposure (no, yes). The community level variables were place of residence (urban, rural), distance to health facility (big problem, not a big problem), community literacy level (low, medium, high) and community socioeconomic status (low, medium, high).

### Statistical analyses

First, descriptive analysis was performed using frequency and percentage distributions to examine respondents’ sociodemographic characteristics and health insurance enrollment. Second, bivariate logistic regression was applied to select the explanatory variable and covariates that had a significant association with health insurance enrollment with p-value less than 0.05 as a cut-off point. Results were presented as crude odds ratios (COR). Third, a multicollinearity test was performed using variance inflation factor (VIF) to check for collinearity among selected variables. The test found no evidence of collinearity among the explanatory variables (Mean VIF = 2.35, Min VIF = 1.0, Max VIF = 5.39). VIF less than 10 are tolerable [41]. In the final step, four different models were constructed using multilevel logistic regressions (MLLR) to assess whether individual/household and community level factors had significant associations with the outcome variables (health insurance enrollment). The first model was a null/empty model (Model 0), which did not have the explanatory variable or covariates, attributed to the primary sampling unit (PSU). The second model (Model I) comprised individual-level factors and the third model (Model II) comprised community-level factors. The last model (Model III) was the complete model that included both the individual/household and community-level factors. Results were presented as adjusted odds ratios (AOR).

All four MLLR models included fixed and random effects [42]. The fixed-effects model showed the association between all included variables and the outcome variable, and the random effects showed the measure of variation in the outcome variable based on PSU, which was measured by Intra-Cluster Correlation Coefficient (ICC) [43]. The model fit was assessed using Akaike’s Information Criterion (AIC) [44]. We used the “melogit” command to run the MLLR models. The “svyset” command was used to account for survey weight, cluster and strata. The analyses were performed using Stata version-14 software (Stata Corp, College Station, Texas, USA). We also followed the guidelines for Strengthening Observational studies in Epidemiology (STROBE) [45].

### Ethical clearance

We used publicly available secondary data for this study (available at: https://dhsprogram.com/data/available-datasets.cfm). Ethical procedures were ensured by the institutions that funded, commissioned, and managed the surveys. No further ethical clearance was required. Additional details about data and ethical standards are available at http://goo.gl/ny8T6X.

## Results

### Background characteristics of respondents

A total of 226,734 married women aged 15–49 were included in this study. One fifth (20.2%) were young between the ages of 15 to 24 years. More than three-fourth (76.3%) of the respondents were from male headed households. About 30.5% and 13.3% of respondents were not currently employed and had no media exposure, respectively. About 60.5% of respondents were rural residents, and 24.4% reported they encountered a big problem when visiting a health facility. About 55.9% of the respondents had decision-making autonomy either alone or together with their husbands on all three decision-making parameters: their own health, to make large household purchases, and to visit families/relatives (Table 2).

### Coverage of health insurance

**Fig. 1:**
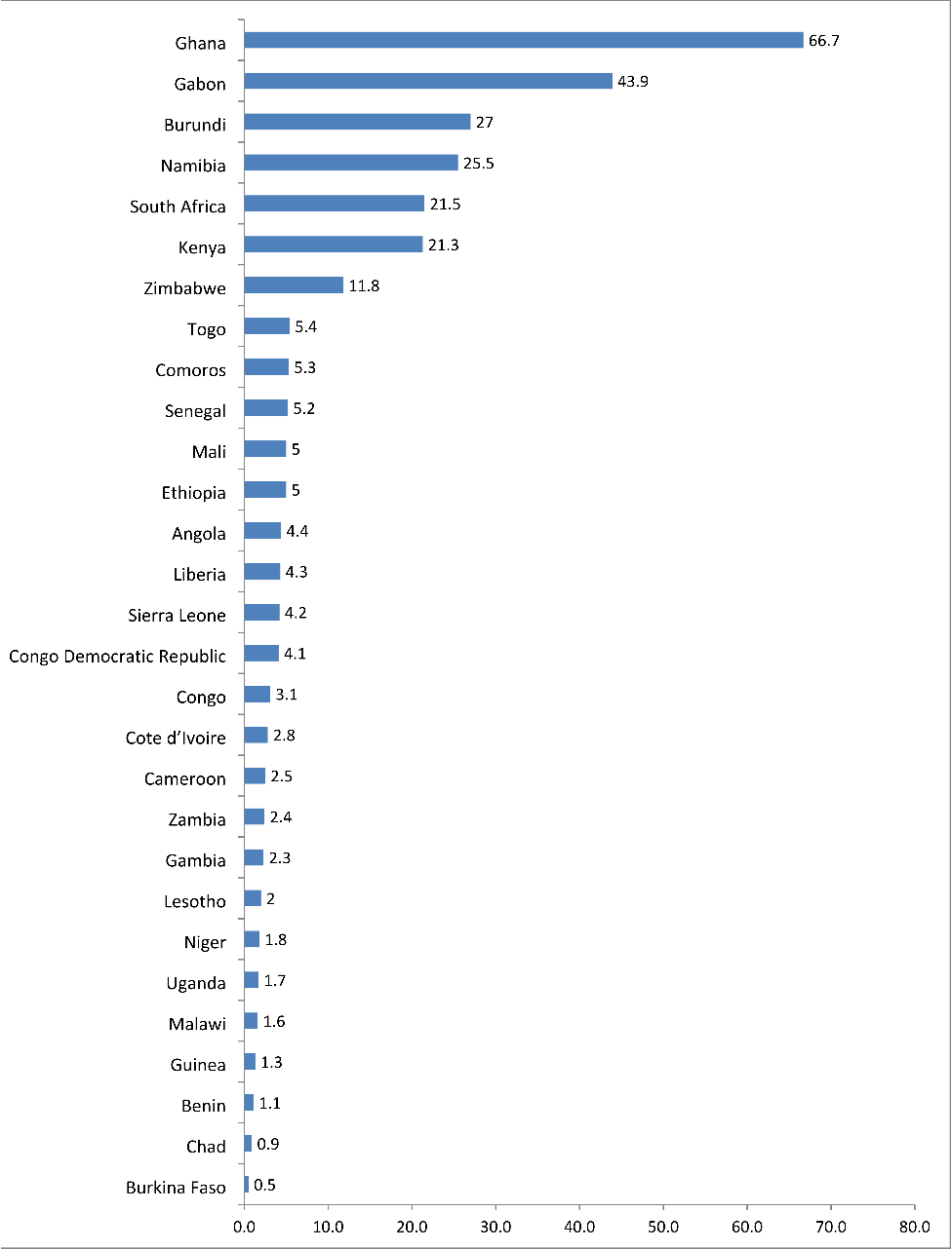
Coverage of health insurance among married women: Evidence from 29 sub-Saharan African countries Demographic and Health Surveys (N = 226,734)

The pooled results showed that about 21.3% of married women were covered by health insurance (Table 2). The lowest coverage was observed in Burkina Faso (0.5%), Chad (0.9%) and Benin (1.1%) respectively, while the highest coverage was seen in Ghana (66.7%), Gabon (43.9%) and Burundi (27.0%) respectively (Fig. 1).

### Distribution of health insurance across explanatory variable and covariates

Health insurance coverage varied from 15.7% among women who had no decision-making autonomy to 25.8% among those who had decision-making autonomy. Coverage also varied from 4.9 to 26.0% between adolescent women (15–19 years) and older women (40–44 years). We observed significant difference in health insurance coverage across subgroups of educational level among women and their husbands. For instance, health insurance coverage was 2.7% among women who had no formal education and about 68.1% among women who had higher education. Similarly, health insurance coverage varied from 2.4% among women whose husbands had no formal education to 58.9% among women whose husbands had higher education. The coverage of health insurance also varied from 3.0 to 44.3% between women from the poorest and richest households respectively. Health insurance coverage was 3.7% among women who had no media exposure and 24.0% among those who had media exposure. We further observed that coverage varied from 15.7 to 29.8% between women who lived in rural and urban areas, respectively (Table 2).

**Table 2:** Frequency distribution of respondents, distribution of health insurance across explanatory variables and bivariate results: Evidence from 28 sub-Saharan African countries Demographics and Health Surveys (N = 226,734)

Variable	Frequency (Weighted %)	Health Insurance coverage (%)	COR (95% CI)
**Overall health insurance coverage**	226,734 (21.3%, 95% CI; 19.9-22.7%)		
**Women’s decision-making autonomy**			
No	140,633 (44.1)	15.7	Ref
Yes	96,376 (55.9)	25.8	1.86 (1.48–2.33)***
**Women’s age in years**			
15–19	15,695 (3.40)	4.90	Ref
20–24	40,061 (16.8)	13.7	2.62 (1.23–5.61) *
25–29	48,465 (24.9)	22.5	5.75 (2.73–12.12) ***
30–34	41,715 (19.7)	23.2	5.42 (2.60-11.28) ***
35–39	35,622 (15.6)	25.0	6.94 (3.24–14.82) ***
40–44	25,598 (10.5)	26.0	8.44 (3.87–18.37) ***
45–49	19,578 (8.70)	22.0	6.54 (3.04–14.09) ***
**Women’s educational level**			
No formal education	98,811 (9.10)	2.7	Ref
Primary school	70,054 (54.3)	12.3	4.19 (2.22–7.89) ***
Secondary school	50,548 (27.0)	29.3	12.59 (6.61–23.95) ***
Higher	7,292 (9.40)	68.1	96.37 (48.09-193.12) ***
**Husband’s educational level**			
No formal education	82,697 (6.60)	2.4	Ref
Primary school	57,030 (48.0)	11.0	3.53 (1.26–9.83) *
Secondary school	64,301 (31.4)	24.7	8.87 (3.13–25.12) ***
Higher	15,830 (13.7)	58.9	48.95 (17.11-139.99) ***
** Wealth status**			
Poorest	52,109 (16.7)	3.0	Ref
Poorer	47,328 (18.0)	8.2	3.01 (1.78–5.08) ***
Middle	44,346 (19.0)	14.9	8.22 (4.91–13.76) ***
Richer	41,570 (21.6)	25.8	22.60 (13.17–38.77) ***
Richest	41,381 (24.5)	44.3	75.66 (42.56-134.53) ***
**Parity**			
**1–2**	32,719 (16.5)	26.2	Ref
3–4	100,153 (55.6)	24.3	1.03 (0.77–1.38)
5+	78,577 (27.8)	11.6	0.56 (0.39–0.80)**
**Sex of household head**			
Male	188,798 (76.3)	20.8	Ref
Female	37,936 (23.7)	22.9	1.78 (1.39–2.27)***
**Currently employed**			
No	81,801 (30.5)	12.5	
Yes	144,798 (69.4)	25.2	2.65 (2.04–3.45)***
**Media exposure**			
No	78,479 (13.3)	3.7	Ref
Yes	147,962 (86.6)	24.0	5.85 (3.53–9.70)***
**Family size**			
< 5	68,479 (46.16)	25.6	Ref
>=5	158,255 (53.8)	17.6	0.87 (0.71–1.07)
**Place of residence**			
Urban	73,668 (39.5)	29.8	Ref
Rural	153,066 (60.5)	15.7	0.28 (0.22–0.36)***
**Distance to health facility**			
Big problem	96,421 (24.4)	11.4	Ref
Not a big problem	130,272 (75.5)	24.5	1.63 (1.25–2.12)***
**Community literacy level**			
Low	88,161 (25.8)	8.9	Ref
Medium	76,206 (35.1)	20.0	3.94 (2.92–5.32)***
High	62,367 (39.0)	30.6	10.86 (8.05–14.65)***
**Community socioeconomic status**			
Low	122,808 (44.5)	10.5	Ref
Medium	38,563 (15.5)	21.5	4.77 (3.57–6.38)***
High	65,363 (39.8)	33.3	9.44 (7.33–12.17)***

### Fixed effect (measure of association)

The odds of health insurance coverage was higher among women who had decision-making autonomy (AOR = 1.33, 95% CI; 1.03–1.72) compared to women who had no decision-making autonomy. Women in the age groups of 35–39 years (AOR = 3.02, 95% CI; 1.01–9.05) and 40–44 years (AOR = 3.78, 95% CI; 1.22–11.71) had higher odds of health insurance coverage compared to adolescents aged 15–19 years. The results showed higher odds of health insurance coverage among women who had secondary (AOR = 3.29, 95% CI; 1.48–7.35) and higher education (AOR = 10.62, 95% CI; 4.37–25.81) compared to those who had no formal education. Similarly, women whose husbands had higher education (AOR = 4.32, 95% CI; 1.40-13.33) were more likely to have health insurance coverage compared to those whose husbands had no formal education. We observed higher odds of health insurance coverage among women who were from the middle (AOR = 3.57, 95% CI; 2.01–6.33), richer (AOR = 6.19, 95% CI; 3.37–11.36) and richest (AOR = 11.64, 95% CI; 5.68–23.82) households compared to woman from the poorest households. Women who were employed (AOR = 2.19, 95% CI; 1.60-3.00) had higher odds of health insurance coverage than women who were not employed. Women who were exposed to media (AOR = 1.77, 95% CI; 1.05-3.00) were more likely to be covered than women who were not. We further found higher odds of health insurance coverage among women from female headed household (AOR = 1.48, 95% CI; 1.13–1.94) compared to those from male headed households. Regarding community level, higher odds of health insurance coverage was observed among women who belonged to medium community socioeconomic status (AOR = 1.79, 95% CI; 1.27–2.54) compared to those belonging to low community socioeconomic status (Table 3).

**Table 3:** Fixed effect results for the association between women’s decision-making autonomy and health insurance enrollment among married women: Evidence from 29 sub-Saharan African countries Demographics and Health Surveys (N = 226,734)

Variable	Model I	Model II	Model III
Decision-making autonomy			
No	Ref		Ref
Yes	1.33 (1.03–1.72)*	1.83 (1.46–2.29)***	1.33 (1.03–1.72)*
**Women’s age**			
15–19	Ref		Ref
20–24	1.17 (0.41–3.34)		1.15 (0.40–3.28)
25–29	2.04 (0.70–5.92)		2.01 (0.69–5.80)
30–34	2.10 (0.73–6.04)		2.06 (0.72–5.91)
35–39	3.10 (1.03–9.28)*		3.02 (1.01–9.05)*
40–44	3.88 (1.25-12.00)*		3.78 (1.22–11.71)*
45–49	2.51 (0.81–7.74)		2.44 (0.79–7.51)
**Women’s educational level**			
No formal education	Ref		Ref
Primary school	1.84 (0.86–3.94)		1.73 (0.80–3.74)
Secondary school	3.51 (1.59–7.73)**		3.29 (1.48–7.35)**
Higher	11.42 (4.76–27.36)***		10.62 (4.37–25.81)***
**Husband educational level**			
No formal education	Ref		Ref
Primary school	1.12 (0.37–3.40)		1.12 (0.36–3.43)
Secondary school	1.73 (0.55–5.41)		1.72 (0.54–5.45)
Higher	4.33 (1.41–13.24)*		4.32 (1.40-13.33)*
** Wealth status**			
Poorest	Ref		Ref
Poorer	1.75 (0.98–3.12)		1.66 (0.94–2.94)
Middle	3.83 (2.15–6.81)***		3.57 (2.01–6.33)***
Richer	6.75 (3.71–12.26)***		6.19 (3.37–11.36)***
Richest	12.70 (6.51–24.80)***		11.64 (5.68–23.82)***
**Parity**			
One	Ref		Ref
2–4	1.25 (0.81–1.95)		1.26 (0.81–1.97)
5+	0.69 (0.39–1.20)		0.70 (0.40–1.22)
**Currently employed**			
No	Ref		Ref
Yes	2.20 (1.61–3.01)***		2.19 (1.60-3.00)***
**Media exposure**			
No	Ref		Ref
Yes	1.78 (1.05–3.01)*		1.77 (1.05-3.00)*
**Sex of household head**			
Male	Ref		Ref
Female	1.47 (1.12–1.93)**		1.48 (1.13–1.94)**
**Distance to health facility**			
Big problem		Ref	Ref
Not a big problem		1.52 (1.16–1.99)**	1.20 (0.89–1.62)
**Place of residence**			
Urban		Ref	Ref
Rural		0.99 (0.76–1.28)	1.25 (0.93–1.68)
**Community literacy level**			
Low		Ref	Ref
Medium		2.43 (1.80–3.29)***	1.06 (0.73–1.55)
High		4.20 (3.07–5.73)***	1.47 (1.00-2.15)*
**Community socioeconomic status**			
Low		Ref	Ref
Medium		3.07 (2.28–4.14)***	1.79 (1.27–2.54)**
High		4.54 (3.33–6.19)***	1.13 (0.75–1.70)

### Random effect (measure of variation)

The random effect models of married women’s decision-making autonomy and health insurance are shown in Table 4. We observed that the values of the AIC decreased across the models, indicating a best-fitted model. The ICC in the null model (ICC = 0.59) showed that the odds of health insurance varied across clusters (σ2 = 4.81, 4.14–5.58). The between-cluster variations decreased by 7% in Model I, from 59% in the null model to 52% in Model I. From Model I, the ICC decreased again by 2% in Model II (ICC = 0.50) and then again increased by 2% in the complete model (Model III, ICC = 0.52). These estimates showed that the variations in the likelihood of health insurance can be attributed to the variances in the clustering at the primary sampling units (Table 4).

**Table 4:** Random effect results for the association between married women’s decision-making autonomy and health insurance: Evidence from 28 sub-Saharan African countries Demographics and Health Surveys (N = 226,734)

Random effect	Model 0	Model I	Model II	Model III
PSU variance (95% CI)	4.81 (4.14–5.58)	3.67 (3.08–4.38)	3.28 (2.79–3.85)	3.65 (3.07–4.34)
ICC	0.59	0.52	0.50	0.52
Wald chi-square and p-value	Ref	χ2 = 398.44, p < 0.001	χ2 = 390.58, p < 0.001	χ2 = 518.64, p < 0.001
**Model fitness**				
Log-likelihood	-12242.85	-9159.73	-11897.726	-9140.49
AIC	24489.71	18367.47	23813.45	18340.98
N				

Notes: *p < 0.05; **p < 0.01; **p < 0.001; Ref = reference category; AIC = Akaike Information Criterion; PSU = Primary Sampling Unit; N = total observation; ICC = Intra-class correlation coefficient

## Discussion

In this study, we examined the association between women’s decision-making autonomy and health insurance coverage among married women in SSA using Demographic and Health Survey datasets of 29 countries in Africa. The pooled results showed that health insurance coverage was approximately 21.3%. The lowest coverage was seen in Burkina Faso (0.5%), Chad (0.9%) and Benin (1.1%) respectively, while highest coverage was observed in Ghana (66.7%), Gabon (43.9%) and Burundi (27.0%) respectively.

Our study found that women with decision-making autonomy had a higher chance of obtaining health insurance than women without decision-making autonomy. This finding is consistent with prior studies where highly autonomous women were noted to have high self-esteem and may not accept gender power differences [46]. There is evidence that health care is critical in reducing maternal and child morbidity and mortality [46–49]. Although women often make decisions regarding primary health care, a majority do not have health insurance to cover their families [47]. Increasing healthcare costs combined with slow income growth have led to losses in health insurance for women and an increased rate of barriers to accessing needed care and paying medical bills [50]. Educational programs and awareness-raising initiatives aimed at women as informed healthcare users and decision-makers are becoming increasingly important [51]. According to the Pew Internet Project, more and more, women are turning to social media for health care information, to share their health care experiences, as well as make decisions and recommendations, connecting with other women in similar situations [52]. Additionally, women in decision-making positions in health care are known to be trusted sources of health information [51].

Achieving gender equality and empowering all women and girls is the main focus under the seventeen “Sustainable Development Goals” set for transforming the world and improving quality of life [53].

In order to achieve the proposed “Sustainable Development Goals” through appropriate interventions, there is no doubt about the importance of focusing on the position of women in decision-making to address the issue of gender inequality through design of strategies and adoption of immediate intervention measures to empower women [54–56].

Married women in older age groups had a higher chance of health insurance coverage compared to adolescents; this is consistent with prior studies in Ghana [57, 58], Nigeria [59], Kenya, Nigeria and Tanzania [3] and Ethiopia [31, 60]. Evidence suggests that older women believe they are more susceptible to diseases as they age [31]. As older age groups become more concerned with their health - and because they are more likely to be employed - they will be more likely to afford or pay for insurance [31].

The findings revealed higher odds of health insurance coverage for married women who had secondary and tertiary education compared to those who had no formal education. Studies in Western Ethiopia and Senegal [61, 62], Nigeria [63], and Ethiopia [29, 64] had similar findings. A possible explanation may be that educated people may understand the principles of health insurance systems and benefit packages [29]. Educated people may be well informed about services and gain a better understanding of their benefits, which drives them to sign up with health insurance schemes. This further suggests that raising women’s awareness through education campaigns can contribute to the successful implementation of health insurance schemes [29].

Likewise, we also found that husband’s educational level associated with coverage of health insurance as seen in prior studies in Nepal and Nigeria [65, 66]. This may be because an educated person understands the principles of health insurance and benefits package [65]. Evidence shows that respondents with higher awareness of health insurance had more chance to be enrolled into health insurance [67]. This is because informed individuals are likely to seek more information about the service, and a better understanding of the benefits of encouraging participation in the CBHI community can contribute to the success of the program [68].

In contrast with previous studies in Ethiopia suggesting that low- and moderate-wealth households were more likely than high-wealth households to enroll in health insurance systems [29, 69, 70], our study showed higher odds of health insurance coverage among women from the wealthiest households compared to the poorest ones. A plausible explanation may be the poor implementation of health care policies which leads wealthy households to prefer to pay -out of pocket for immediate health care services instead of waiting for care through services covered by health insurance [3, 71].

The odds of having health insurance coverage were higher for married women who were employed. This might be due to employment opportunities which enable women to meet specific health needs [72, 73]. It might also be due to employed women being insured by their employer [74]. Women reinvest up to 90% of their income in the health and education of their families, so considering women’s needs and preferences is not only a social investment, but also makes economic sense [74]. Women, especially working mothers, want their parents, spouses, and children to be insured [74]. This finding suggests that encouraging women to take up jobs may improve their socioeconomic status, which may be beneficial to their health and well-being [75].

As previously observed in SSA women exposed to media were more likely to have health insurance compared to those with no media exposure [3]. Evidence suggests that people who listen to radio, watch television, or read newspapers are more likely to sign up for public health insurance because they know and understand the benefits and importance of having coverage [3]. This highlights a central role that the media plays in the dissemination of health-related knowledge and strategies since media is recognized as a powerful tool for successful dissemination and acceptance of health measures [76, 77].

This study further revealed higher odds of health insurance enrollment by the women in female-headed households compared to male-headed households. Preliminary insights into the relationship between gender and healthcare decision-making can be gleaned from the literature on healthcare-seeking behavior, in which several reviews have examined the intersection between home and family roles [78, 79]. A systematic review by Colvin et al. (2013) on health-oriented behavior found that the timely treatment of sick household members is inextricably linked to the degree of influence that the mother has on the final decision to seek external help [79]. Health risk assessments have been shown to manifest differently in male and female householders due to their unique household roles in the study setting. Therefore, it is postulated that women prioritize their direct knowledge of household health needs when deciding to join a health insurance scheme [78]. Existing literature recognizes that the burden of care falls primarily on women, a dynamic that is particularly pronounced when disease occurs in the home setting [79, 80]. This underlying knowledge of the physical, psychological, and economic costs of illness makes the female voice a necessity for understanding and assessing health risks in the household [79].

Higher odds of health insurance enrollment were seen among women of medium community socioeconomic status compared to those of low socioeconomic status. This underscores the impact of systems that provide opportunities for people from a better socioeconomic community to contribute and enroll in health insurance [32].

## Strengths and limitations of the study

This study has several strengths. First, the study contributes to the body of knowledge by filling in the gaps on the relationship between women’s decision-making ability and health insurance enrollment in SSA. Second, the study was a cross-country analysis with a large and nationally representative sample. Therefore, the results of this study are generalizable to several sub-Saharan African countries and can be used by policy makers and program planners to improve health insurance coverage. However, our results must be interpreted in the context of the following limitations. First, the cross-sectional nature of the study does not allow the conclusion of a cause-and-effect relationship. Second, since the study was based on self-reported information, memory bias could affect the results. Third, the present study was limited to married women only and therefore cannot be generalized for all women of childbearing age. Finally, and due to data limitations, this study relied on surveys that were collected at different points in time, however, evaluation studies suggest that these differences do not affect the comparability of the data [81].

## Conclusion

This study revealed that health insurance coverage is low among married women in SSA. The findings suggest that enhancing women’s autonomy and socioeconomic status, as well as improving media exposure and community socioeconomic conditions, are crucial in promoting health insurance enrollment. Given the critical role of health insurance in ensuring access to healthcare services, it is imperative for policymakers to prioritize the empowerment of women in SSA through various interventions such as education, financial support, and community development programs. Our study highlights the need for targeted health policies aimed at improving health insurance coverage among married women in SSA. By addressing the identified factors that influence health insurance enrollment, these policies can lead to improved health outcomes, reduced healthcare costs, and ultimately, sustainable development in the region.

## Data Availability

The datasets generated and/or analyzed during the current study are available in DHS Program – available datasets [82].
